# *Achromobacter xylosoxidans* Infections after Prostate Biopsies, France, 2014

**DOI:** 10.3201/eid2511.161487

**Published:** 2019-11

**Authors:** Lucie Amoureux, Julien Bador, Catherine Neuwirth

**Affiliations:** University Hospital of Dijon, Dijon, France

**Keywords:** *Achromobacter* spp., nosocomial infections, species identification, reservoir, resistance, bacteria, France

**To the Editor:** We read with interest the article by Haviari et al. concerning a health care–associated outbreak of *Achromobacter xylosoxidans* infections after prostate biopsies (*1*). Although noteworthy, the description lacks some data.

First, the isolates of *Achromobacter* cannot be referred to as *A. xylosoxidans* from just the method used in this study, API 20 NE mass spectrometry (bioMérieux, https://www.biomerieux.com). Since 2012, a total of 18 species have been defined in the genus *Achromobacter* (*2*). Only multilocus sequence typing or sequencing 765 bp of the housekeeping gene *nrdA* enables the identification of the isolates to the species level (*3*). To date, in the few studies available, a great variety of species have been detected in clinical samples, with *A. xylosoxidans* the most predominant (*4**,**5*). Correct identification of the isolates involved in all types of infection is necessary to help understand the epidemiology, pathogenicity, and resistance pattern of the various species.

Second, the antimicrobial drug resistance profiles are not given (except for ceftriaxone, which is an intrinsic resistance, and ofloxacin) but again are valuable epidemiologic data. This information might help in detecting the emergence of new cases in the unit or in other hospitals, as well as in discussing the therapeutic options.

Finally, all the bacteria recovered in the container belonged to environmental waterborne genera frequently encountered in wet sites in hospitals. As discussed by the authors, these microorganisms have been involved in contamination of antiseptic solutions containing quaternary ammonium compounds or chlorhexidine. Unfortunately, the authors did not mention which disinfectants were used in the biopsy room (for hands, sinks, surfaces, or containers) and did not investigate for these potential sources of contamination. In the absence of identification of any reservoir and despite the new measures adopted, new cases might still occur.

In conclusion, these missing data are needed for other hospitals to identify epidemiogenic *Achromobacter* isolates. Complete information would help in implementing control measures to contain and prevent outbreaks.
